# Supplementary material for: Air monitoring by nanopore sequencing
Source: ISME Commun. 2024 Jul 16;4(1):ycae099. doi: 10.1093/ismeco/ycae099 (PMC11287864; doi:10.1093/ismeco/ycae099)
Supplement: Supplementary_Information_final_ycae099 [file supplementary_information_final_ycae099.pdf]

Supplementary Information

Supplementary Table 1. Sampling, DNA, and sequencing data of all air samples (*pilot_study* and *urban_study* sheets): sampling data includes date, temperature, humidity, and sampling duration; DNA metrics include total yield and yield per m^3; and sequencing data includes total number of reads, filtered reads, read length distribution (median and N50), read-based taxonomic classification results using Kraken2, and assembly statistics using metaflye (Materials and Methods).

Supplementary Table 2. Environmental pollution of urban sampling location measured through particle mass fractions (TSP, PM10, and PM2.5; TSP=total suspended particles; PM=particulate matter); measurements were taken in one-minute intervals (Materials and Methods).

Supplementary Table 3. Antimicrobial resistance and virulence genes detected by ABRicate and AMRFinderPlus across all air samples (*pilot_study* and *urban_study* sheets) with respective gene coverage and mapping accuracy metrics (Materials and Methods).

Supplementary Figure 1. Taxonomic composition on the taxonomic phylum level using Kraken2, Diamond, and CZID annotations (Materials and Methods) of the **A.** controlled (Gh), and and **B.** natural (Nat) environment air samples.

Supplementary Figure 2. Taxonomic composition on the taxonomic genus level using Kraken2, Diamond, and CZID annotations (Materials and Methods) of the **A.** controlled (Gh), and and **B.** natural (Nat) environment air samples.

Supplementary Figure 3. Relative abundance of microbial genera detected in the negative control samples from **A.** the controlled (Gh) and **B**. natural (Nat) environments (1: sampling control; 2: extraction control; 3: sequencing control). **C.** Relative expected versus observed relative abundance of microbial species in the positive control sample from a defined mock community (Materials and Methods).

Supplementary Figure 4. Particle mass fraction measurements across urban sampling locations for **A.** TSP, **B.** PM_{10}, and **C.** $PM_{2.5}$ [$\mu g/m^3$]. The p-values describe the differences between all locations using the Kruskal-Wallis test (Materials and Methods).

Supplementary Figure 5. Analysis of COG functional categories in controlled (Gh) and natural (Nat) air samples. **A.** Annotation of assembled contigs. **B.** Annotation of MAGs. Each bar aggregates all functionalities detected across the respective samples from the same sampling condition within the same functional category.

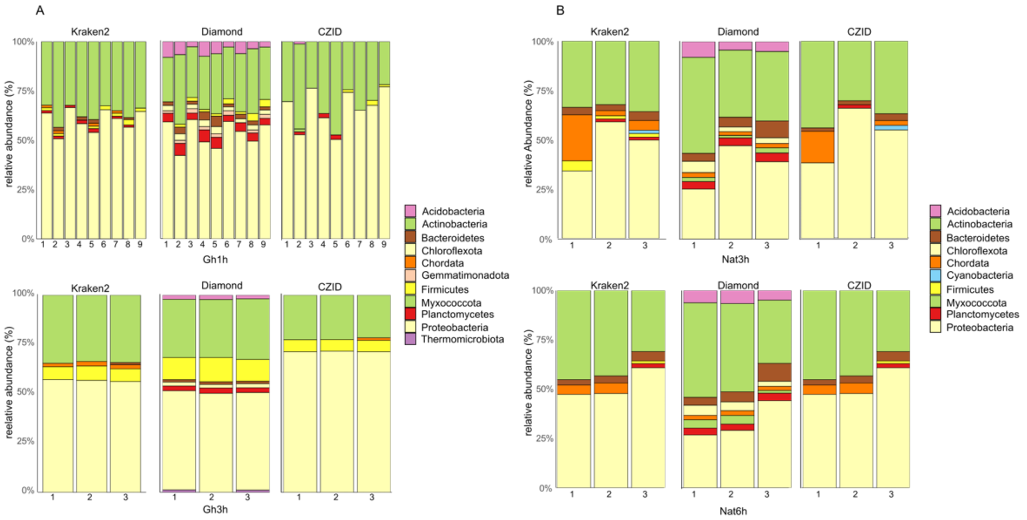
A
B
Kraken2
Diamond
CZID
Gh1h
Gh3h
Nat3h
Nat6h
relative abundance (%)
relative Abundance (%)
100%
75%
50%
25%
0%
Acidobacteria
Actinobacteria
Bacteroidetes
Chloroflexota
Chordata
Gemmatimonadota
Firmicutes
Myxococcota
Planctomycetes
Proteobacteria
Thermomicrobiota
Cyanobacteria

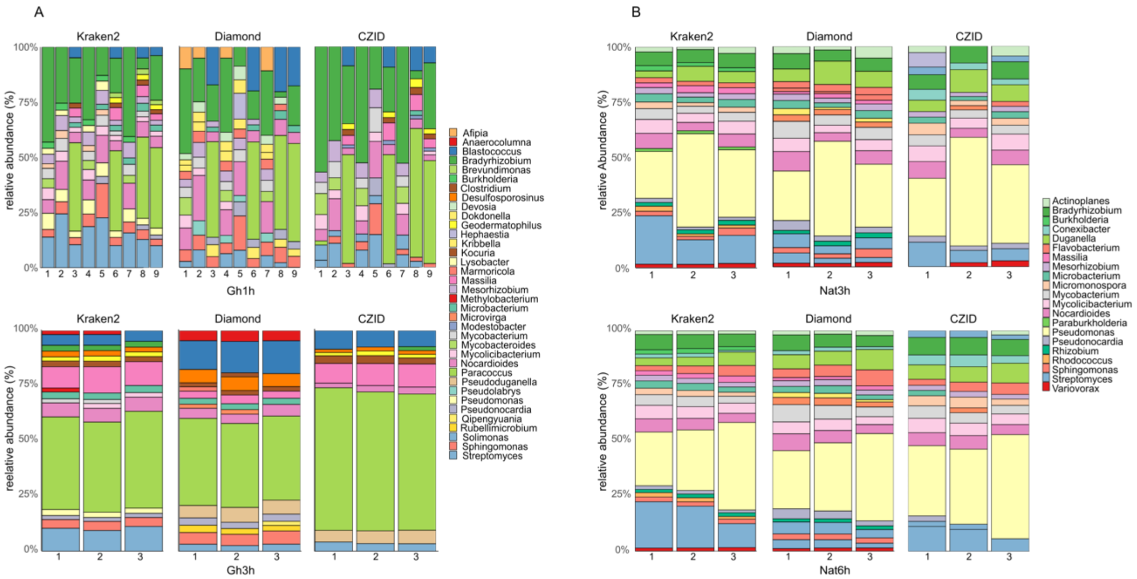
A
B
Kraken2
Diamond
CZID
Gh1h
Gh3h
Nat3h
Nat6h
relative abundance (%)
relative Abundance (%)
Afipia
Anaerocolumna
Blastococcus
Bradyrhizobium
Brevundimonas
Burkholderia
Clostridium
Desulfosporosinus
Devosia
Dokdonella
Geodermatophilus
Hephaestia
Kribbella
Kocuria
Lysobacter
Marmoricola
Massilia
Mesorhizobium
Methylobacterium
Microbacterium
Microvirga
Modestobacter
Mycobacterium
Mycobacteroides
Mycolicibacterium
Nocardioides
Paracoccus
Pseudoduganella
Pseudolabrys
Pseudomonas
Pseudonocardia
Qipengyuania
Rubellimicrobium
Solimonas
Sphingomonas
Streptomyces
Actinoplanes
Conexibacter
Duganella
Flavobacterium
Micromonospora
Paraburkholderia
Rhizobium
Rhodococcus
Variovorax

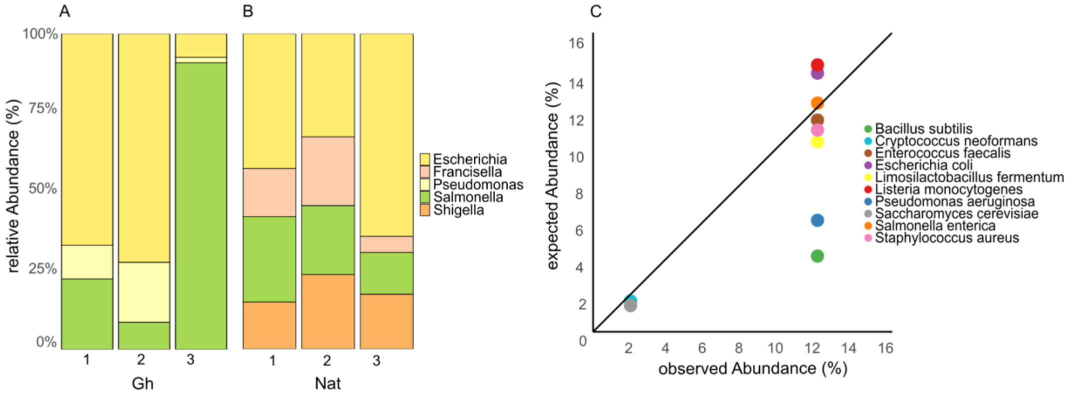
A
B
C
relative Abundance (%)
100%
75%
50%
25%
0%
Gh
Nat
Escherichia
Francisella
Pseudomonas
Salmonella
Shigella
expected Abundance (%)
observed Abundance (%)
Bacillus subtilis
Cryptococcus neoformans
Enterococcus faecalis
Escherichia coli
Limosilactobacillus fermentum
Listeria monocytogenes
Pseudomonas aeruginosa
Saccharomyces cerevisiae
Salmonella enterica
Staphylococcus aureus

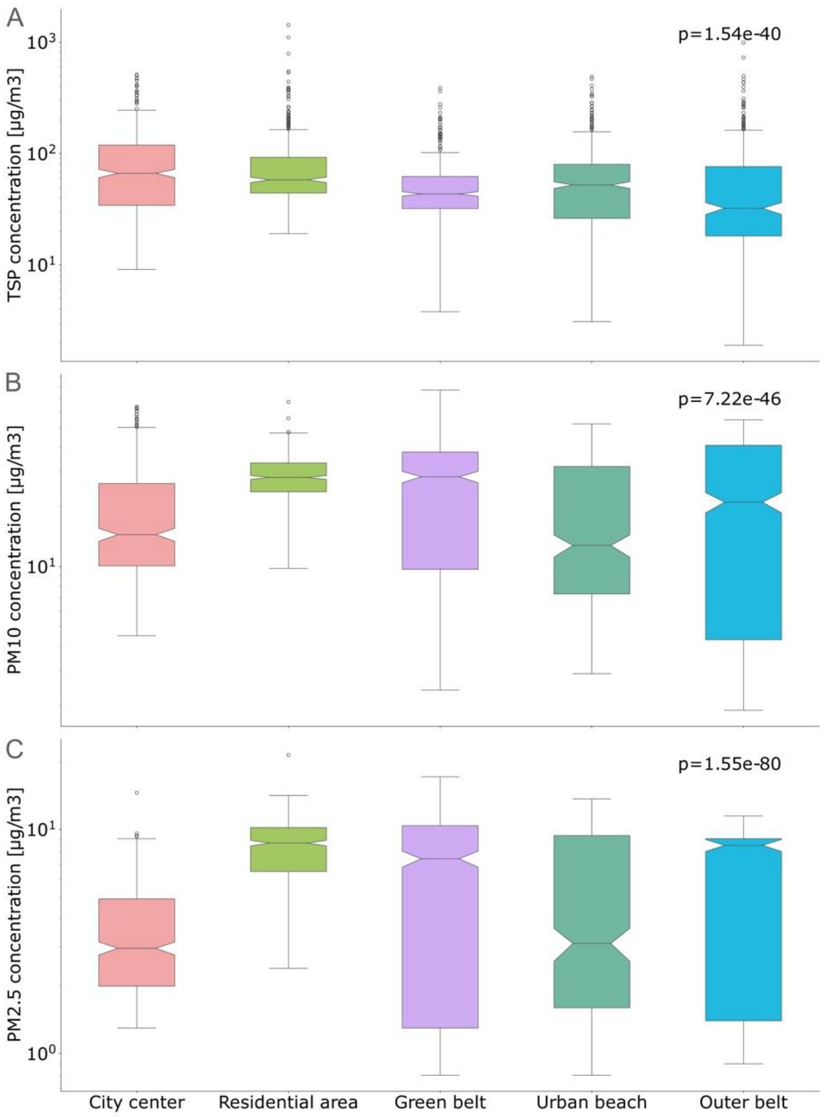
A
B
C
p=1.54e-40
p=7.22e-46
p=1.55e-80
TSP concentration [µg/m3]
PM10 concentration [µg/m3]
PM2.5 concentration [µg/m3]
City center
Residential area
Green belt
Urban beach
Outer belt

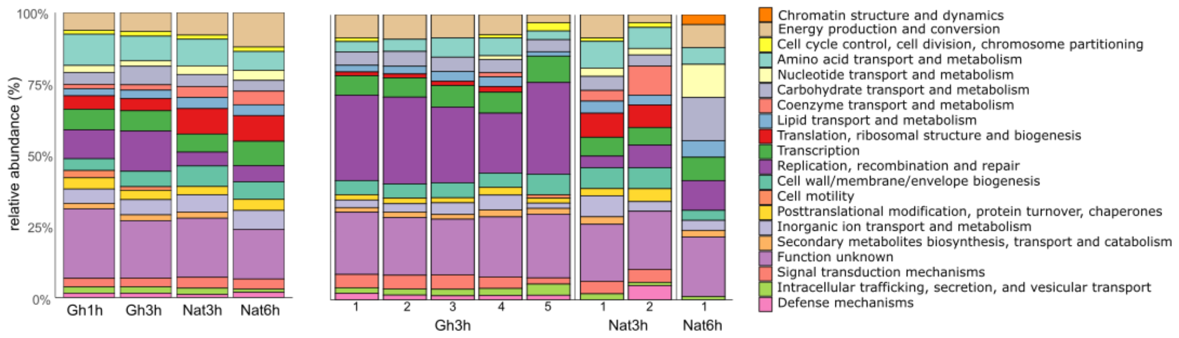
relative abundance (%)
100%
75%
50%
25%
0%
Gh1h
Gh3h
Nat3h
Nat6h
1
2
3
4
5
Chromatin structure and dynamics
Energy production and conversion
Cell cycle control, cell division, chromosome partitioning
Amino acid transport and metabolism
Nucleotide transport and metabolism
Carbohydrate transport and metabolism
Coenzyme transport and metabolism
Lipid transport and metabolism
Translation, ribosomal structure and biogenesis
Transcription
Replication, recombination and repair
Cell wall/membrane/envelope biogenesis
Cell motility
Posttranslational modification, protein turnover, chaperones
Inorganic ion transport and metabolism
Secondary metabolites biosynthesis, transport and catabolism
Function unknown
Signal transduction mechanisms
Intracellular trafficking, secretion, and vesicular transport
Defense mechanisms

Air sampling and DNA extraction optimizations

We first tested two standard air sampling approaches, the high-volume sampler (HVS, MCV, Spain) and the Coriolis µ liquid impinger (Bertin Technologies, France), to assess the optimal air sampler for their compatibility with nanopore shotgun sequencing.

For the HVS, we used quartz filters for air sampling for 24h at a rate of 500 L/min. We applied both, phenol-chloroform extraction [1] and the standard PowerSoil Pro kit (QIAGEN, 2018), to the filters. While the phenol-chloroform method resulted in a higher total DNA yield than the standard extraction kit (data not shown), the nanodrop nucleic acid 260/280 measurements of around 1.2 indicated that the extracted DNA was highly contaminated, most likely due to residual phenol, which would block the nanopores during shotgun sequencing. The DNA yield of the standard extraction kit, on the other hand, was not sufficient for nanopore shotgun sequencing, which made us hypothesize that the standard kit – since not optimized for DNA extractions from quartz filter – might have chemically enhanced binding of the particles to the silica-enriched filters, and might therefore have made extraction inefficient.

For the liquid impingement-based and therefore filter-free sampler Coriolis µ, we sampled air for 1h at a rate of 300 L/min, and extracted sufficient DNA using the standard Qiagen kit: To increase DNA concentrations, we benchmarked that the volume of the final elution buffer (EB) could be reduced from the

standard of 50 µL to 30µL. We further tested if a repeated washing of the spin column would further increase the DNA yield, which was not the case:

**Sample #**	**Volume EB**	**DNA concentration (ng/µL)**	**Total DNA (ng)**
1	50 µL	0.131	6.55
2	50 µL	0.283	14.15
3	50 µL	0.330	16.50
4	30 µL	1.240	37.2
5	30 µL	0.767	23.01
6	30 µL	1.69	50.7
7	30 µL twice	0.607	18.09
8	30 µL twice	0.627	18.93
9	30 µL twice	0.609	18.48

Functional annotation

The general functional analysis of the *de novo* assemblies and MAGs revealed a broad spectrum of COG (Clusters of Orthologous Genes) functional categories in our controlled and natural air samples. Briefly, gene predictions were made using Prodigal v2.6.3 [2], with COG functional categories analyzed using eggNOG v2.0.1 [3] and taxonomically classified using DIAMOND BLASTP. As eggNOG lacked taxonomic resolution, we also applied Prokka v1.14.6 [4] followed by DIAMOND BLASTP to the bins, which delivered taxonomic and functional annotation. Following the findings in 'Omics Insights in

Environmental Bioremediation', we filtered the annotated gene list to select genes involved in biodegradation and bioremediation. For comparing the functional inferences between the different sampling durations and locations, we calculated the relative abundance of the functional categories for the contigs (**Supplementary Figure 5A**) and MAGs (**Supplementary Figure 5B**) across samples of each experiment. We found a broad spectrum of genes encompassing diverse COG functional categories. The gene distribution was relatively similar between the controlled and the environmental setting, which was expected given the very basic metabolic and replication functionalities that are being described by COG.

The functional annotation of MAGs further allowed us to predict taxon-specific functions of the air microbiome. We, for example, obtained a *de novo* assembly of *Sphingomonas alba*, which has previously only been defined through a soil isolate [5] and might therefore represent a novel strain with important functional variation. Our genome annotation identified genes (*flr, ribBA*) from flavin-based metabolic cycles, and a gene (*cher1*) which plays a role in biofilm formation and chemotaxis [6]. Certain bacterial taxa exhibit chemotactic responses towards aromatic hydrocarbons, which are prevalent pollutants, since they utilize these compounds as carbon sources; the *cher1* gene has been identified as a key gene in mediating this behavior [7].

1. Castro JF, Nouioui I, Sangal V, Trujillo ME, Montero-Calasanz MDC, Rahmani T, et al. Geodermatophilus chilensis sp. nov., from soil of the Yungay core-region of the Atacama Desert, Chile. *Syst Appl Microbiol* 2018; **41**: 427–436.
2. Hyatt D, Chen G-L, LoCascio PF, Land ML, Larimer FW, Hauser LJ. Prodigal: prokaryotic gene recognition and translation initiation site identification. *BMC Bioinformatics* 2010; **11**: 1–11.
3. Huerta-Cepas J, Szklarczyk D, Heller D, Hernández-Plaza A, Forslund SK, Cook H, et al. eggNOG 5.0: a hierarchical, functionally and phylogenetically annotated orthology resource based on 5090 organisms and 2502 viruses. *Nucleic Acids Res* 2019; **47**: D309–D314.
4. Seemann T. Prokka: rapid prokaryotic genome annotation. *Bioinformatics* 2014; **30**: 2068–2069.
5. Siddiqi MZ, Rajivgandhi G, Lee S-Y, Im W-T. Characterization of four novel bacterial species of the genus Sphingomonas, Sphingomonas anseongensis, Sphingomonas alba, Sphingomonas brevis and Sphingomonas hankyongi sp.nov., isolated from wet land. *International Journal of Systematic and Evolutionary Microbiology* 2023; **73**: 005884.
6. García-Fontana C, Reyes-Darias JA, Muñoz-Martínez F, Alfonso C, Morel B, Ramos JL, et al. High Specificity in CheR Methyltransferase Function: CheR2 OF PSEUDOMONAS PUTIDA IS ESSENTIAL FOR CHEMOTAXIS, WHEREAS CheR1 IS INVOLVED IN BIOFILM FORMATION*. *Journal of Biological Chemistry* 2013; **288**: 18987–18999.

7. Lacal J, Muñoz-Martínez F, Reyes-Darías J-A, Duque E, Matilla M, Segura A, et al. Bacterial chemotaxis towards aromatic hydrocarbons in Pseudomonas. *Environmental Microbiology* 2011; **13**: 1733–1744.
